# Decision-making approaches for children with life-limiting conditions: results from a qualitative phenomenological study

**DOI:** 10.1186/s12910-022-00788-7

**Published:** 2022-05-16

**Authors:** Sidharth Vemuri, Jenny Hynson, Katrina Williams, Lynn Gillam

**Affiliations:** 1 Victorian Paediatric Palliative Care Program, The Royal Children’s Hospital Melbourne, 50 Flemington Road, Parkville, VIC 3052 Australia; 2 Victorian Paediatric Palliative Care Program, The Royal Children’s Hopsital Melbourne, 50 Flemington Road, Parkville, VIC 3052 Australia; 3 Department of Paediatrics, Monash University, 246 Clayton Road, Clayton, VIC 3168 Australia; 4 Children’s Bioethics Centre, The Royal Children’s Hospital Melbourne, 50 Flemington Road, Parkville, VIC 3052 Australia

**Keywords:** Decision making, Parents, Life-limiting illness, Paediatric palliative care, Family-centred care, Qualitative research

## Abstract

**Background:**

For children with life-limiting conditions who are unable to participate in decision-making, decisions are made for them by their parents and paediatricians. Shared decision-making is widely recommended in paediatric clinical care, with parents preferring a collaborative approach in the care of their child. Despite the increasing emphasis to adopt this approach, little is known about the roles and responsibilities taken by parents and paediatricians in this process. In this study, we describe how paediatricians approach decision-making for a child with a life-limiting condition who is unable to participate in decision-making for his/herself.

**Methods:**

This qualitative phenomenological study involved 25 purposively sampled paediatricians. Verbatim transcripts from individual semi-structured interviews, conducted between mid-2019 and mid-2020, underwent thematic analysis. Interviews were based around a case vignette matched to the clinical experience of each paediatrician.

**Results:**

Two key themes were identified in the exploration of paediatricians' approach to decision-making for children with life-limiting conditions: (1) there is a spectrum of paediatricians’ roles and responsibilities in decision-making, and (2) the specific influences on paediatricians’ choice of approach for end-of-life decisions. In relation to (1), analysis showed four distinct approaches: (i) non-directed, (ii) joint, (iii) interpretative, and (iv) directed. In relation to (2), the common factors were: (i) harm to the child, (ii) possible psychological harm to parents, (iii) parental preferences in decision-making, and (iv) resource allocation.

**Conclusions:**

Despite self-reporting shared decision-making practices, what paediatricians often described were physician-led decision-making approaches. Adopting these approaches was predominantly justified by paediatricians’ considerations of harm to the child and parents. Further research is needed to elucidate the issues identified in this study, particularly the communication within and parental responses to physician-led approaches. We also need to further study how parental needs are identified in family-led decision-making approaches. These nuances and complexities are needed for future practice guidance and training around paediatric decision-making.

*Trial registration:* Not applicable.

**Supplementary Information:**

The online version contains supplementary material available at 10.1186/s12910-022-00788-7.

## Introduction

Shared decision-making (SDM) aims to protect and maximise the autonomy of adult patients and is often espoused as an ideal for clinicians to work toward [1–3]. While definitions differ, SDM is most commonly thought of as a middle path between physician-led decision-making, where the doctor decides on the treatment after evaluation of the disease, treatment options and probabilities of outcomes, and informed decision-making, where the patient decides after the doctor provides information about the benefits, risks, and alternative treatment options [4]. SDM is more complex in paediatrics because there are three parties involved: the child, parent(s) and clinician [2, 5, 6]. There is lack of clarity in the literature about whether decisions are being shared between the child and clinician, parent and clinician, or between all three. For children with life-limiting conditions (LLC) who are unable to participate in decision-making due to their developmental capacity, decisions are made by their parents and clinicians. In this context, decisions are being made *for* the child rather than *with* the child [2, 7]. Despite this, there is an increasing emphasis on the routine implementation of SDM in paediatric clinical care [2, 3]. Theoretically, SDM aims to facilitate family-centred care [3, 8], but there are few empirical studies examining the relative roles of paediatricians and parents in decision-making, nor details about how, and the extent to which, SDM is used in practice. This study describes how paediatricians approach decision-making for a child with a LLC who is unable to participate in decision-making for him/herself.

## Methods

### Ethical approval

Ethical approval was obtained from The Royal Children’s Hospital Melbourne Human Research Ethics Committee (HREC/50340/RCHM-2019).

### Study design

This qualitative exploration was part of a wider phenomenological study exploring how paediatricians conceptualise advance care planning and prepare families for end-of-life (EOL) decision-making. Phenomenology was particularly well-suited to this study, given it seeks to explore how paediatricians create meaning and understand reality in their lived and subjective experiences of decision-making, focusing on richness of data rather than size of the sample [9, 10].

Paediatricians in Victoria, the second most populous state in Australia, who provide clinical care for children with LLC, excluding those in specialist palliative care teams, were recruited via professional and departmental networks, and snowballing. Purposive sampling ensured inclusion of paediatricians caring for children with neurodisability, cancer and complex cardiac disease, in both acute intensive and long-term service settings. Voluntary written consent was obtained.

### Data collection

Individual semi-structured interviews (45–150 min in duration) were conducted between mid-2019 and mid-2020, audio-recorded and transcribed verbatim, with de-identification. The interviewer was S.V., a trained qualitative researcher and paediatric palliative care physician, who was known to all participants.

Paediatricians’ approaches to one of the five clinical vignettes (Table 1) were explored and used to prompt more general discussion in the interview. Vignettes, developed in accordance with published recommendations [11], were matched to each paediatrician’s clinical experience to improve plausibility [12]. Two vignettes involved a child with severe neurodisability, one in an outpatient clinic and the other in the intensive care unit. Two vignettes involved a child with cancer, one with a solid tumour and the other with a haematological malignancy. The final vignette involved a child with complex congenital heart disease. Face validity of each vignette was confirmed by two independent physicians from international paediatric centres.

**Table 1:** Clinical vignettes to prompt discussion in interviews

*Vignette 1: Child with a severe neurodisability being seen in an outpatient clinic*
Part 1: You are seeing a 7-year-old boy with GMFCS V cerebral palsy of unknown cause, and associated epilepsy who requires gastrostomy feeding. This outpatient clinic review is approximately four weeks after a recent prolonged inpatient admission where he had a serious illness requiring non-invasive ventilatory support (no previous requirement for respiratory support at home) Part 2: His parents have seen on multiple parent blogs about the role of extracorporeal membrane oxygenation (ECMO) in critical illnesses and would like to document their preference for ECMO if he has another serious illness
*Vignette 2: Child with a severe neurodisability admitted in the intensive care unit*
Part 1: You are taking over responsibility for a 7-year-old boy with GMFCS V cerebral palsy of unknown cause, and associated epilepsy who requires gastrostomy feeding. This boy was admitted to PICU one week ago with a serious illness requiring non-invasive ventilatory support that has not been able to be weaned (no previous requirement for respiratory support at home) Part 2: His parents have seen on multiple parent blogs about the role of extracorporeal membrane oxygenation (ECMO) in critical illnesses and would like to document their preference for ECMO during this illness
*Vignette 3: Child with a solid tumour*
Part 1: You are seeing a 5-year-old girl with relapsed, widely metastatic neuroblastoma who is currently well and not on treatment Part 2: Her parents have seen on multiple parent blogs about the role of extracorporeal membrane oxygenation (ECMO) in critical illnesses and would like to document their preference for ECMO if she has another serious illness
*Vignette 4: Child with a haematological malignancy*
Part 1: You are seeing an 8-year-old boy with multiply relapsed AML, who has been diagnosed with a subsequent relapse four months following second HSCT. He is clinically well and not currently on treatment Part 2: His parents have seen on multiple parent blogs about the role of extracorporeal membrane oxygenation (ECMO) in critical illnesses and would like to document their preference for ECMO if he deteriorates
*Vignette 5: Child with complex congenital heart disease*
Part 1: You are due to meet the parents of a 3-month-old baby girl currently on ECMO. Her background includes: Antenatal diagnosis of hypoplastic left heart syndrome IVF conception after 7 years of attempts Underwent Norwood stage 1 procedure at 2 days of age. On return to PICU, she had a rising lactate and escalating inotropes, prompting cannulation for ECMO at 8 h post-operatively Required 5 days of ECMO support before decannulation Two-month admission in PICU before being transferred to the cardiology ward Most recent echocardiogram demonstrated moderately reduced ventricular function with moderate tricuspid valve regurgitation. Two days ago, she had progressive desaturation with a cardiac arrest, and was cannulated onto ECMO after 25 min of CPR Part 2: Her parents have seen on multiple parent blogs about the role of long-term ventricular assist device (VAD) support and transplantation and would like to document their preference for these interventions

Three authors (S.V., J.H., and L.G.) developed an interview guide, which was pilot-tested with K.W. The interview guide included reference to one of the five vignettes, and included prompts to explore paediatricians’ approaches to, and intention of, communication (Additional file 1).

### Data analysis

**Fig. 1:**
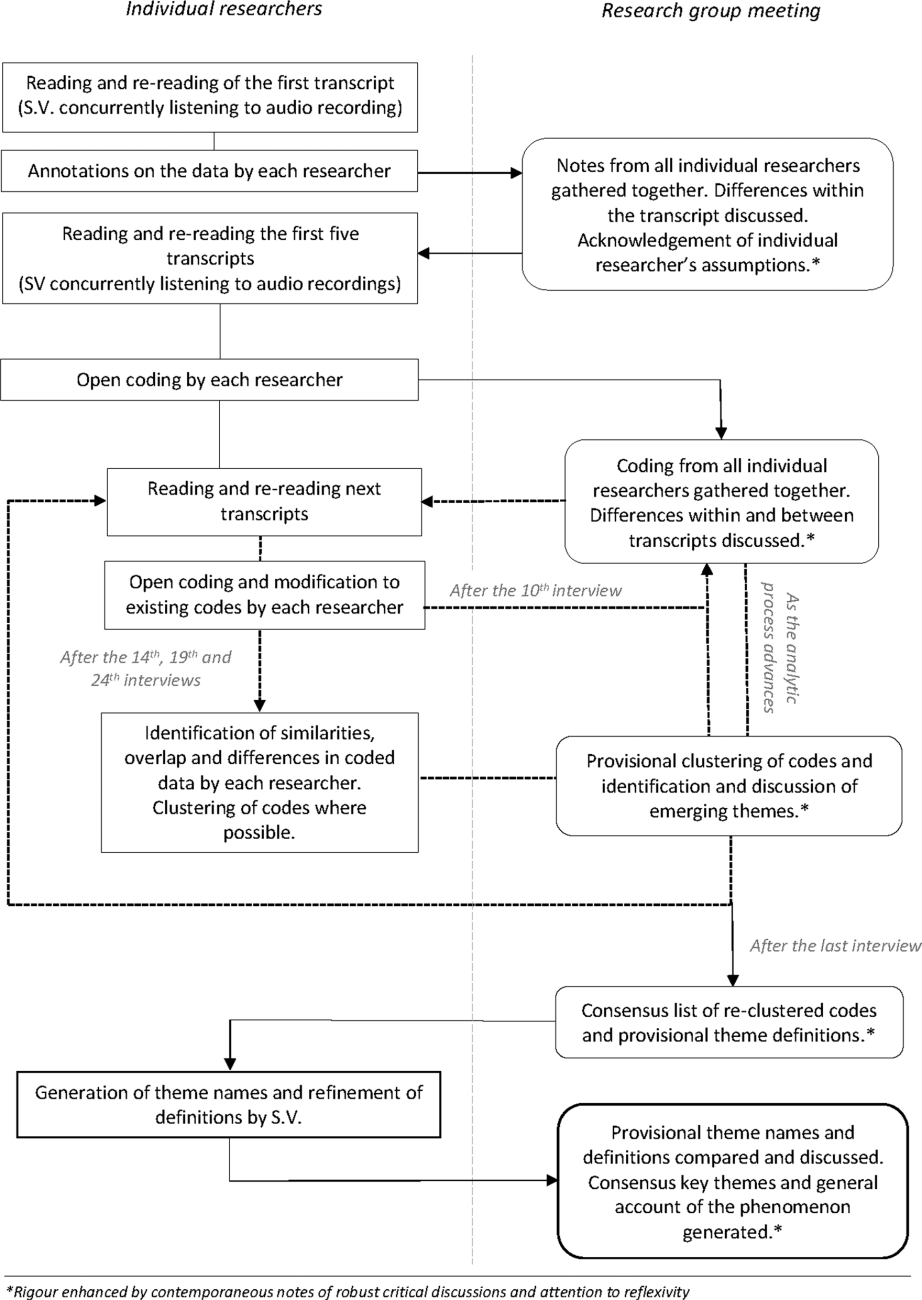
Process of thematic analysis

Data collection and thematic analysis [13] was an iterative process over 13 months (illustrated in Fig. 1); six iterations allowed for clarification of emerging themes and following new lines of inquiry. After reading and re-reading the first transcript, open coding was conducted individually by S.V., J.H. (paediatric palliative care physician and experienced post-doctoral qualitative researcher) and L.G. (clinical ethicist and experienced post-doctoral qualitative researcher), then discussed together and refined. This was repeated after every fifth interview. As the study progressed, codes were grouped by individual researchers, and then discussed and refined by the team to identify emerging themes. Rigour was maintained through prolonged engagement with the data, contemporaneous notes of discussion in the research team meetings, and by attention to reflexivity (using field notes and post-interview debriefs). Contemporaneous notes were also taken of the robust critical discussion between research team members. Data were managed using a combination of hardcopy and electronic NVivo [14] files.

## Results

### Participants

Twenty-five of the 102 approached paediatricians (25%) participated in this study. Demographics are detailed in Table 2. Seventeen paediatricians were male, and 12 had more than 10 years’ experience working at consultant level. Nineteen paediatricians worked in a tertiary paediatric hospital.

**Table 2:** Clinician demographics

Paediatrician	Gender	Subspecialty	Location of work^a^	Experience^b^	Interview mode
*Vignette 1: Child with a severe neurodisability in an outpatient clinic*					
P-01	Male	General	Tertiary/Metropolitan^c^	> 20 years	In-person
P-02	Female	General	Tertiary/Metropolitan^c^	> 20 years	In-person
P-03	Female	General	Tertiary/Metropolitan^d^	5–10 years	In-person
P-04	Female	General	Tertiary/Metropolitan^d^	< 5 years	In-person
P-05	Male	General	Secondary/Metropolitan^d^	16–20 years	In-person
P-06	Female	General	Tertiary/Metropolitan^d^	5–10 years	In-person
P-07	Male	General	Secondary/Rural^d^	16–20 years	In-person
P-08	Male	General	Secondary/Rural^d^	11–15 years	In-person
P-09	Male	General	Secondary/Rural^d^	16–20 years	In-person
P-10	Male	General	Secondary/Rural^d^	> 20 years	In-person
*Vignette 2: Child with a severe neurodisability in an intensive care admission*					
P-11	Male	Intensivist	Tertiary/Metropolitan^c^	5–10 years	In-person
P-12	Male	Intensivist	Tertiary/Metropolitan^c^	16–20 years	In-person
P-13	Male	Intensivist	Tertiary/Metropolitan^c^	5–10 years	In-person
P-14	Female	Intensivist	Tertiary/Metropolitan^c^	< 5 years	In-person
P-15	Female	Intensivist	Tertiary/Metropolitan^c^	> 20 years	Videoconference
*Vignette 3: Child with a haematological malignancy*					
P-16	Female	Oncologist	Tertiary/Metropolitan^c^	< 5 years	In-person
*Vignette 4: Child with a solid tumour*					
P-17	Male	Oncologist	Tertiary/Metropolitan^c^	5–10 years	In-person
P-18	Female	Oncologist	Tertiary/Metropolitan^c^	11–15 years	In-person
P-19	Male	Oncologist	Tertiary/Metropolitan^c^	5–10 years	In-person
*Vignette 5: Child with complex congenital heart disease*					
P-20	Male	Cardiologist	Tertiary/Metropolitan^d^	5–10 years	Videoconference
P-21	Male	Cardiologist	Tertiary/Metropolitan^c^	11–15 years	In-person
P-22	Male	Cardiologist	Tertiary/Metropolitan^c^	16–20 years	Telephone
P-23	Male	Cardiologist	Tertiary/Metropolitan^c^	5–10 years	In-person
P-24	Male	Intensivist	Tertiary/Metropolitan^c^	11–15 years	In-person
P-25	Male	Intensivist	Tertiary/Metropolitan^c^	5–10 years	In-person

^a^Location of work classified by the Department of Health and Human Services, Victorian Government [34]. Tertiary paediatric centres are children’s hospitals with subspecialty departments. Secondary centres are general paediatric departments within an adult hospital

^b^Years’ experience working at consultant level

^c^Public clinical practice only

^d^Combination of both public and private clinical practice

### Key themes

Analysis identified two key themes in relation to paediatricians' approach to decision-making for children with LLC; (1) there is a spectrum of paediatricians’ roles and responsibilities in decision-making, and (2) the specific influences on paediatricians’ choice of approach for EOL decisions.

### Theme 1: Spectrum of roles and responsibilities in decision-making

**Fig. 2:**
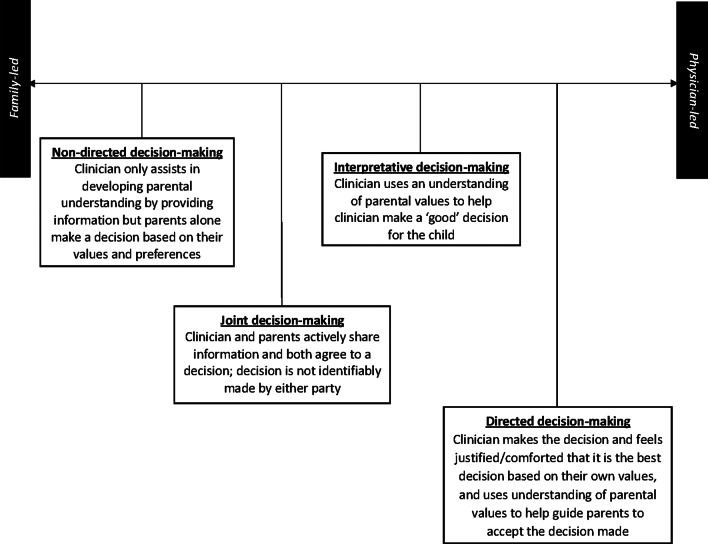
Spectrum of decision-making in paediatrics

There was a spectrum in how paediatricians conceptualised their role in decision-making; within this spectrum four distinct approaches were identified (Fig. 2). Most paediatricians framed their approach as SDM, however, description of their roles and responsibilities often indicated an intentional physician-led process.

#### Non-directed decision-making

At one end of the spectrum, a few paediatricians described a non-directed decision-making approach (which could also be called family-led decision-making). In this approach, paediatricians did not seek to influence the decision nor be the decision-maker but rather convey *“information…[at] the level the family wants…then they [parents] make up their decision themselves”* (P-20) based on *“their values and what they want”* (P-09). Recognising that *“it’s not my child, it’s their child”* (P-05), these paediatricians *“never seek to take away [parental] decision-making"* (P-05) and *“present the range [of management options]…as part of a menu…[and] don’t try and direct [decision-making]”* (P-07). However, most paediatricians were concerned about *“just giving options”* (P-22), because informing parents of *“everything like a buffet [of management options]…is confusing”* (P-16).

#### Joint decision-making

In the middle of the spectrum, some paediatricians described another process, a joint decision-making approach, which was *“a two-way conversation”* (P-11). Acknowledging that parents *“have control and…[know] their child best”* (P-15), these paediatricians marry *“our perception with the parent’s perception to come up with some composite [decision]”* (P-01). The difference between this and other decision-making approaches was that responsibility for this *"joint decision"* (P-11) was not identifiably the parents nor the paediatrician’s as neither party had full responsibility for the outcome of the decision.

#### Interpretative decision-making

Moving towards the physician-led end of the spectrum, some paediatricians described an interpretative approach; they used their inferences about family values to guide their decision-making. One paediatrician identified parental values from *“what they say, by their choices that they make, by the way they interact with their child”* and used this to inform his* “decisions as the patient’s agent”* (P-01). This was supported by another, who described that through *“discussion about what is happening to their child…if you are attuned to the cues, you can usually understand what they [parents] want you to do [without asking them for a decision]”* (P-12). In this approach, paediatricians focused on taking *“responsibility for what happens to the child and not the parents”* (P-02).

#### Directed decision-making

At the extreme physician-led end of the spectrum, a small number of paediatricians, particularly those who provide oncological, cardiac, or intensive care, described a directed decision-making approach where it is their *“duty is to make decisions and guide families”* (P-22) as *“in many cases there isn’t a choice but that is only because in our experience that patient should be cured with this pathway”* (P-18). For one paediatrician: *“there is usually nothing that they [parents] provide me that helps with determining therapy”* (P-17). The responsibility for decision-making at this end of the spectrum varied. In some circumstances, it was held by an individual clinician, in others it was shared by the clinical team. One paediatrician stated that *“I have always taken it that it is my decision to not give somebody paediatric intensive care…I am making that decision”* (P-15). This contrasts with what was described by most paediatricians caring for children with cancer or cardiac disease; *“you sort of wipe your hands of the ultimate responsibility because…this is a team decision”* (P-23). While taking responsibility for decisions was common for paediatricians describing both interpretative and directed approaches, in directed decision-making, this moved beyond taking responsibility to *“taking it [control of the decision] away from them [the family]”* (P-11). For paediatricians adopting this approach, *“providing care is to make the decision…and if you can’t make a decision then you’re not doing your job properly”* (P-22). They viewed sharing the decision as informing the family of the *“rationale for your thinking”* (P-22) but ultimately aimed to *“influence”* (P-09) and *“guide families based on our experience”* (P-23).

Some paediatricians who reported taking this directed decision-making approach described using communication to intentionally guide the family towards the decision they felt would be best for the child; *“you pitch your discussion with them”* (P-22), *“leading it that way, in the hope that they get to that point [decision] before I do”* (P-15). By *“drip feeding them information informally…[then] pitching information in a more formal setting…parents [get] to kind of share in that decision”* (P-15). If parents challenged the directed decision, paediatricians stated that *“it is really important that the family’s opinion is acknowledged…and that you [then] try and redirect them to the reasons why, the pros and cons [of the paediatrician’s decision]”* (P-23) and *“normally they trust you and believe you”* (P-11). All paediatricians who adopted this directed approach acknowledged *“we always couch it as a shared decision”* (P-14).

### Theme 2: Specific influences on paediatricians’ choice of approach for EOL decisions

Decision-making approaches varied not only between paediatricians; individual paediatricians also adopted different approaches depending on the clinical circumstances. For potential EOL decisions, there were four considerations that influenced the approach taken by paediatricians. Physician-led approaches were more commonly adopted in this clinical context.

#### Risk of harm to the child

Most paediatricians described that the *“nature of the decision…how major that decision is, whether it involves a consequence”* (P-09) influenced the decision-making approach adopted. As one paediatrician stated: *“when it is a flip of a coin one way or the other…it’s not such a big deal [if parents take the lead]…[as] I don’t think it makes a big difference to the outcome”* (P-22). However, for decisions with significant outcomes, such as in relation to the child’s EOL, many paediatricians described being well, if not best, placed to make these decisions, noting that *“it’s really hard to separate [best interests of] the child from the family”* (P-04). Paediatricians identified *“the key thing [of decisions] is what is right for the patient, [with] secondary consideration as well of what is right for the parents and family”* (P-06). The responsibility in decision-making is *“to do what you think’s best for the child”* (P-11) as *“decisions are based on what I think is right for the child”* (P-19), *“my role is to make sure the child is not harmed”* (P-02) and *“we have to protect the patient [from harm]”* (P-14). This reflected use of more physician-led decision-making approaches.

Challenges arise when there are differences between what parents and paediatricians think is in the best interests of the child. In the context of continuing or pursuing life-prolonging therapy, where *“if you put a bit more effort in, they [the child] would probably live a bit longer, but you are not necessarily going to do that”* (P-15), paediatricians more often considered harm to the child from continued interventions as opposed to harm from death. One paediatrician expressed: *“when parents put limitations on treatment that seem appropriate, we’re more willing to kind of accept them, but when they’re asking for more intervention…that’s when there’s angst amongst the clinical team”* (P-04). This contrasts with another paediatrician: *“I don’t think we have any right to make a judgement about whether it is in the child’s best interest not to be here…I’ve never regretted giving children a bit more of a chance, a bit more time…[while] some people might say ‘oh that dragged on in the ICU for a long time’, but I don’t think that, because what matters in the end is the family’s feelings…hopefully comforted by that fact that everything possible was done”* (P-12).

Although the threshold of harm to the child from continued interventions is *“hard to pick up”* (P-18), “*there’s always a line that you’re not willing to cross”* (P-06). However, often this is *“a fine and ethically difficult line…[as when] we believe a certain course of treatment is in the child’s best interest, we may not always stress the uncertainty to the family”* (P-01). In these circumstances, some paediatricians assess *“what’s acceptable and what’s not acceptable”* (P-18) for the child. An acceptable decision for the child may not necessarily be what paediatricians consider to be in the child’s best interests and is *“much more open to what families think is appropriate”* (P-24), suggestive of an interpretative approach as opposed to a directed decision-making approach. As one paediatrician summarised: *“knowing that parents are certainly better observers of the child than we are, but also their perception is coloured by their own experience, trying to be objective and to marry our perception with the parent’s perception to come up with some composite that actually is acting in the child’s best interest is extremely difficult and then having decided where that is, trying to negotiate with the family to exclude things that are clearly not in the child’s best interest, to not necessarily persuade the family to our perception but to a position that is acceptable”* (P-01).

When raising disagreement about a choice of intervention due to its potential to harm the child, some paediatricians were concerned about establishing an *“adversarial relationship”* (P-14), while others felt disempowered: *“society is not ever going to fight against the desire of a parent to do what they want to do, sometimes beyond the best interest of the child…it has nothing to do with just medicine”* (P-25).

#### Perceived psychological harm to the parents

Outside consideration of harm to the child at EOL, paediatricians also described the influence of perceived psychological harm to parents from this decision-making. Paediatricians described EOL decision-making as an *“incredibly emotionally difficult place to be in”* (P-10) and recounted *“seeing the distress and the inability of parents to make a decision”* (P-01). Such experiences influenced most paediatricians to take more physician-led approaches: *“the idea that parents can decide when is the right time for their child to die is really hard…you’re too enmeshed, you’re too distressed, you’re too emotionally challenged”* (P-02), so *“you have to make the decision, you have to take it away from them [parents] because for that family at that point, it is horrific”* (P-11). However, in contrast, one paediatrician observed that with these decisions, *“we feel guilty and don’t want to have that responsibility…[so] we allow too much burden of decision making to lay in the family’s hands”* (P-13), intimating awareness of a shift in approach to non-directed decision-making at these times, albeit one that the paediatrician believed inappropriate.

#### Parental preferences in decision-making

A small number of paediatricians suggested that the decision-making approach was influenced by parental preferences for specific roles and responsibilities. Mirroring the decision-making approaches described in theme 1, one paediatrician described a spectrum of parental preferences for decision-making: *“some [parents] like to be directed and they just want to be told what they should do…[others] weigh up the relative risks and really will give you a response based on what they want…others will meet you halfway”* (P-09). Parental preferences for decision-making approaches were identified informally by observing parents *“making smaller decisions which inform, sometimes [approaches to] bigger decisions”* (P-09), or overtly: *“I will put it on the table and say, ‘I am happy to bear the burden of this decision for you’…they either welcome that or [don’t]”* (P-13).

#### Resource allocation

Resource allocation within the healthcare system was another consideration influencing decision-making approach. Some paediatricians, particularly those caring for children with complex congenital heart disease, viewed themselves as *“the gate keepers of resources and costings to the medical profession…[making] a decision that is in the best interest of not only the family but the hospital, the community, the people who are paying taxes, the people who are donating organs”* (P-23). Their role was to *“say actually no…we are not going to do anything [further]”* (P-23). By working in such clinical contexts which rely on a finite resource, such as heart transplantation, these paediatricians more commonly practised directed decision-making because *"if they [national transplant service] say no, that’s it, all bets are off, there is no recourse”* (P-24).

## Discussion

This exploratory study suggests that SDM can be interpreted and hence practised in different ways. The four approaches to decision-making identified in this study show parallels to relationship models between patients and physicians previously described [15], but contextualised in relation to parental autonomy when approaching decision-making for a child with a LLC. We found that paediatricians accept a joint approach, but only up to a certain point. While SDM is considered the ideal approach to paediatric decision-making [2, 3, 8], our results highlight the complexity involved in SDM for a child with a LLC. Indeed, this finding highlights the complexities of SDM more generally because it raises the question of what is needed for a decision to count as “shared”. Are physician-led approaches best seen as forms of SDM or as totally separate forms of decision-making? Parental preferences for SDM are well-described in the literature: parents want to participate [6, 8, 16, 17], and work collaboratively with paediatricians [8]. This improves overall satisfaction with care [6, 18–23]. Our results suggest that paediatricians feel skilled in giving parents the impression that they have shared in making a decision that was already made. Does it matter if paediatricians lead decision-making provided parents feel like they are involved in the decision-making process? We propose that the important question is not the definitional question of whether physician-led approaches are a form of SDM, but rather the more fundamental ethical question: are physician-led approaches ever ethically permissible in paediatric practice? In what follows, we will identify the two key ethical justifications that our participants alluded to when describing physician-led decision-making, and suggest that they can be valid in the context of decision-making for a child with a LLC. We will also indicate a note of caution about using these justifications.

Our findings, echoed in the literature [6, 17], suggest that many paediatricians believe that physician-led decision-making, which is only partially or not at all based on parents’ initial preferences and values, is sometimes ethically the right way to make decisions about a child’s treatment. The motivation for this appears to come from paediatricians’ protective instincts in two ways: to protect the child from harm and to protect the parents from the psychological burden and possible ongoing harm of making a very difficult decision. In essence, this conceptualises the paediatrician’s role as a guardian [15]. In relation to protecting the child, current ethical models of decision-making support using the consideration of harm to the child as the marker of whether or not to accept parental decisions [24–26]. A parental preference or decision that is not actively harmful to a child should be accepted, even if it may not be the best decision for the child from the paediatrician’s perspective. Yet if parents are wanting something for their child which the paediatrician believes crosses the boundary into harm, then physician-led decision-making, ranging from gentle persuasion to seeking legal intervention, would be ethically appropriate [24–26].

However, paediatricians’ motivation to protect parents from the perceived potential harm of the burden of decision-making is more ethically ‘grey’. We acknowledge the noble intentions behind this motivation, and the wealth of evidence-informed and experiential knowledge paediatricians bring to this decision-making. However, we believe the ethical warrant for protecting adults from being involved in something that will be harmful to themselves, is weaker than the warrant to protect the child from harm that would be caused by someone else (in this case, by well-meaning parents). Protecting adults from harm they might do to themselves, without asking them if they want to be protected, is paternalism, which is now regarded as ethically problematic [15]. This paternalistic desire to protect implies that paediatricians think they know better than parents what is best for them. We suggest that considerable caution is needed in using physician-led decision-making when the prime reason is to protect the parents from the burdens of decision-making.

Parental needs and expectations in decision-making may vary [8]. We recognise that a one-size-fits-all approach does not work and because of this we also caution against assuming that all parents do want to take on the burdens of full responsibility for decision-making in these challenging situations. We propose that the paediatrician’s role is to identify what type of decision is needed in the child’s clinical care and when, and then evaluate where the threshold of harm to the child in relation to this decision lies. If this threshold is not crossed, then the paediatrician should determine and respond to the needs of parents (and how much they choose to participate in decision-making). If there is an unacceptable potential for harm to the child, the paediatrician is ethically obligated to adopt a physician-directed decision-making approach.

A second question that arises from paediatricians’ accounts of physician-led decision-making is this: why couch these decisions as shared with parents? If the paediatrician is taking responsibility for the decision, why do paediatricians not make this overt to parents? By creating an impression that these decisions are shared, paediatricians could be criticised as simply manipulating parents to agree with their intended decision [27]. However, we suggest there is more nuance to this. It may be the case that creating this impression helps parents in their acceptance of their child’s condition, their anticipatory grief around their child’s EOL and improves their outcomes in bereavement, at the same time achieving the decision that best serves the child’s interests. On the other hand, it is also possible that creating this impression may pose a risk for conflict between parents and the treating team, if parents are led to believe that they have an equal say in decisions, and then find that they have no say when they disagree with the paediatrician’s intended decision. Such conflict would be detrimental to the psychological well-being of the parents and the child [19–21, 23]. More data is needed to understand how paediatricians use communication at these times and the possible effects on parents of this aspect of physician-led decision-making. Further ethical thinking is required on the justifiability of leading parents to think they are sharing decision-making when they are not. In addition, further study of family-led decision-making approaches is needed, as much is still unknown about how parental needs are explored by paediatricians at these times, and how family values are inferred. Furthermore, the extent to which parents influence the decision-making approach, and their preferences in communicating their needs and values requires elucidation.

### Limitations

Paediatricians’ participation and self-reported communication practices may have been influenced by their familiarity with S.V., however, this influence, would likely reflect perceived best practice, and differences in responses between paediatricians were still captured. While ‘only’ 25 paediatricians were included this sample size is not considered a true limitation given its consistency with the adopted phenomenological methodological approach [10]. To enhance the reliability of the results, data triangulation could be considered [28]. However, direct observation of discussions around decision-making is ethically and logistically challenging. Clinical simulation offers an alternative to triangulate data related to clinician-specific determinants to decision-making discussions [19, 29–33], and is currently underway.

## Conclusions

Paediatricians in our study reported SDM practices, but their descriptions of their roles and responsibilities indicate different approaches, some of which (interpretive and directed) lie at the physician-led end of the decision-making spectrum. Paediatricians determined the approach at clinical decision-making points and were predominantly influenced by potential harm to the child. Irrespective of parental preferences, many paediatricians felt justified shifting to physician-led decision-making approaches for grave decisions, but intentionally used communication to make parents feel they had been involved in the decision-making process. Further research is needed to explore how paediatricians identify parental needs in family-led decision-making approaches, and how they use and frame nuanced communication within physician-led approaches. Knowledge of how parents respond to these discussions, including their perceptions of being led to believe they shared in decisions that were already made, is also needed. In so doing, the complexities of SDM in paediatric practice could be better understood and incorporated into guidance and training, in the hope of ensuring potential harm to the child is prevented whilst simultaneously promoting child/family satisfaction in care.

## Supplementary Information

**Additional file 1**: Interview guide

## Data Availability

Datasets generated and/or analysed that are not otherwise included in this published article are available from the corresponding author on reasonable request. Deidentified individual participant data will not be made available.

## Abbreviations

SDM: Shared decision-making
LLC: Life-limiting conditions
EOL: End of life
