# Supplementary material for: Decision-making approaches for children with life-limiting conditions: results from a qualitative phenomenological study
Source: BMC Med Ethics. 2022 May 16;23:52. doi: 10.1186/s12910-022-00788-7 (PMC9112587; doi:10.1186/s12910-022-00788-7)
Supplement: Supplementary file 1 — Additional file 1: Interview guide [file 12910_2022_788_MOESM1_ESM.docx]

Interview guide

| **Demographics**   - Details about clinical practice and career experience - Involvement in clinical skills and communication skills training |
| --- |
| **Clinical vignette (see details below)**  *Following Part 1:*   - How would you speak to the family in this clinical situation? - Why would you speak to them with that approach? - What are you hoping to achieve in the consultation? - Are there any concerns or aspects that arise or may arise in the consultation that you would find challenging?   *Following Part 2:*   - How would you respond to this information and request? - What are you hoping to achieve with your response? |
| **Definition and reported practice of ACP**   - Do you feel that your practice in these situations are similar to or different from your colleagues? - Do you think that you would regard this as ACP? - How do you define ACP? - How do you think ACP should go? - How do you train or encourage your Fellows and Registrars to approach these discussions? |
| **Opportunities for subsequent phases of the wider research project**   - How and when could these interactions be observed in a research study? - Do you think these interactions could be audio recorded, observed in person, or both in a research study? |
